# *Pseudomonas fluorescens* Transportome Is Linked to Strain-Specific Plant Growth Promotion in Aspen Seedlings under Nutrient Stress

**DOI:** 10.3389/fpls.2017.00348

**Published:** 2017-03-21

**Authors:** Shalaka Shinde, Jonathan R. Cumming, Frank R. Collart, Philippe H. Noirot, Peter E. Larsen

**Affiliations:** ^1^Biosciences Division, Argonne National LaboratoryLemont, IL, USA; ^2^Department of Biology, West Virginia UniversityMorgantown, WV, USA; ^3^Department of Bioengineering, University of Illinois at ChicagoChicago, IL, USA

**Keywords:** aspen, computational modeling, nitrogen, phosphorus, plant growth promotion, *Pseudomonas*, transportomics

## Abstract

Diverse communities of bacteria colonize plant roots and the rhizosphere. Many of these rhizobacteria are symbionts and provide plant growth promotion (PGP) services, protecting the plant from biotic and abiotic stresses and increasing plant productivity by providing access to nutrients that would otherwise be unavailable to roots. In return, these symbiotic bacteria receive photosynthetically-derived carbon (C), in the form of sugars and organic acids, from plant root exudates. PGP activities have been characterized for a variety of forest tree species and are important in C cycling and sequestration in terrestrial ecosystems. The molecular mechanisms of these PGP activities, however, are less well-known. In a previous analysis of *Pseudomonas* genomes, we found that the bacterial transportome, the aggregate activity of a bacteria's transmembrane transporters, was most predictive for the ecological niche of Pseudomonads in the rhizosphere. Here, we used *Populus tremuloides* Michx. (trembling aspen) seedlings inoculated with one of three *Pseudomonas fluorescens* strains (Pf0-1, SBW25, and WH6) and one *Pseudomonas protegens* (Pf-5) as a laboratory model to further investigate the relationships between the predicted transportomic capacity of a bacterial strain and its observed PGP effects in laboratory cultures. Conditions of low nitrogen (N) or low phosphorus (P) availability and the corresponding replete media conditions were investigated. We measured phenotypic and biochemical parameters of *P. tremuloides* seedlings and correlated *P. fluorescens* strain-specific transportomic capacities with *P. tremuloides* seedling phenotype to predict the strain and nutrient environment-specific transporter functions that lead to experimentally observed, strain, and media-specific PGP activities and the capacity to protect plants against nutrient stress. These predicted transportomic functions fall in three groups: (i) transport of compounds that modulate aspen seedling root architecture, (ii) transport of compounds that help to mobilize nutrients for aspen roots, and (iii) transporters that enable bacterial acquisition of C sources from seedling root exudates. These predictions point to specific molecular mechanisms of PGP activities that can be directly tested through future, hypothesis-driven biological experiments.

## Introduction

Forest ecosystems are major components of the biosphere and contribute extensive ecosystem services. Trees form a significant storage sink in the global carbon (C) cycle, facilitate water fluxes in the hydrologic cycle, and provide wood and fiber for human consumption. The provision of these benefits depends upon the supply and utilization of resources (carbon dioxide, water, nutrients, and light) to and by the tree. However, photosynthesis and primary productivity are often limited by nutrient availability (Houlton et al., 2008; Reich et al., 2009; St. Clair et al., 2009), which, in turn, affects the ecological roles and economic output of forests.

The plant rhizosphere hosts a large and diverse community of microbes whose interactions with roots and soils influence ecosystem productivity (Lambers et al., 2009; Morgan et al., 2010; Cumming et al., 2015).

Interaction between roots and plant growth promoting (PGP) bacteria specifically play critical roles in enhancing the acquisition of nutrients from soils for the plant host and enhance host niche breath and stress resistance (Rodriguez and Fraga, 1999; Barea et al., 2005; Compant et al., 2010). While some aspects of the resource exchange underlying PGP interactions have been described (Morgan et al., 2005; Lambers et al., 2009; Frey-Klett et al., 2011), an increased understanding of these interactions would enhance potential management applications, such as biofuel feedstock production, soil remediation, and C sequestration activities.

*Pseudomonas fluorescens* is commonly found soil bacteria and abundant operational taxonomic units (OTUs) in *Populus* rhizosphere samples (Gottel et al., 2011; Brown et al., 2012). Many *P. fluorescens* strains are categorized as plant growth promoting rhizobacteria (PGPR; Cook et al., 1995). These benefits may be accrued from changes in nutrient availability in the rhizosphere, alterations in host hormonal balance increasing stress resistance, and/or changes in the community of microbes interacting with plant roots (Frey-Klett et al., 2011; Cumming et al., 2015). Some proposed molecular mechanisms for PGP activity include the export of organic acids and siderophores that facilitate dissolution of P in soils (Kurek et al., 2013; Giles et al., 2014; Pastor et al., 2014) and the secretion of phosphatases and phytases that mineralize organic P in the plant rhizosphere (Giles et al., 2014; Pastor et al., 2014). *Pseudomonas* strains have nutrient environment-dependent PGP effects on a variety of crop and tree species, including *Solanum lycopersicon* (Pastor et al., 2014), *Nicotiana tabacum* (Giles et al., 2014), *Oryza sativa* (Habibi et al., 2014), and *Malus domestica* (Kurek et al., 2013). These strain-specific effects on plants can be, in significant part, attributable to differences in *Pseudomonas* transportomes (Silby et al., 2009; Larsen et al., 2015). Transportome is defined here as the relative capacity to transport specific substances across the cell membrane as a function of the set of transmembrane transporters in the genome. An investigation of these differential transportomic capacities of PGP strains and of their correlation with measured phenotypic and biochemical parameters of *Populus tremuloides* seedlings would highlight potential molecular mechanisms underlying the rhizosphere community's specific contributions to plants' acclimation to stress, C sequestration capacity, and the maintenance of productivity under suboptimal conditions.

We have evaluated the effect of four different strains of *Pseudomonas* on *P. tremuloides* (trembling aspen, “aspen”) seedlings in replete media and under conditions of low nitrogen (N) and low phosphorus (P). The evolutionarily distinct *Pseudomonas* strains we chose for this study were Pf0-1, Pf-5, SBW25, and WH6 (Paulsen et al., 2005; Silby et al., 2009; Kimbrel et al., 2010; Loper et al., 2012). Sixteen phenotypic measures of aspen seedlings were collected and computational models of *Pseudomonas* metabolism related to each strain genome were generated. By identifying significant correlations between seedling phenotype colonized by a *Pseudomonas* strains with strain-specific transportomic capacities under different nutrient stress conditions, we were able to predict molecular mechanisms of strain and nutrient stress-specific PGP.

## Methods

### Aspen seedling and *Pseudomonas* resources

*P. tremuloides* Michx. and strains of *P. fluorescens* were cultured together in a laboratory model system. Aspen seeds were obtained from the National Tree Seed Center, Natural Resources Canada, Fredericton NB, Canada. The four *Pseudomonas* strains used in this study were *P. protegens* Pf-5 [ATCC (American Type Cell Culture) Catalog# BAA-477], *P. fluorescens* Pf0-1 (Garbeva et al., 2011), *P. fluorescens* SBW25 (Preston et al., 2001), and *P. fluorescens* WH6 (Banowetz et al., 2008).

### Aspen seedling-*Pseudomonas* vertical plate cultures

Aspen seedling-*Pseudomonas* communities and non-inoculated aspen controls were grown in a vertical plate system under three nutrient regimes: replete, low nitrogen (N), and low phosphorus (P). Experiments were performed using 12 replicate vertical plates per treatment and 8 aspen seedlings per plate for a total of 180 plates and 1,440 aspen seedlings. All data are reported as averages of seedling values from each replicate vertical plate.

Aspen seeds were surface sterilized by washing once with 2% Tween and 2.5% sodium hypochlorite followed by several washes of sterile water. Sterilized seeds were soaked in sterile water in the dark overnight and germinated in jars (Sigma product # V8630) containing 1% Phytablend (Caisson Laboratories, Inc.) for 10 day. Jars were maintained in a growth chamber with 14/10 h light/dark cycle with photosynthetically active radiation (PAR) of 200 μmol m^−2^ s^−1^, temperature regime of 25/20°C, and constant 75% relative humidity before being transferred to the vertical plates (Petri Dishes 150 × 15 mm).

Following the 10 d germination period, seedlings were transferred to a vertical plate system with one of three nutrient treatments. Replete media contained 4 mM NH_4_NO_3_,1 mM CaSO_4_, 1.5 mM K_2_SO_4_, 0.5 mM MgSO_4_, and 1.5 mM KH_2_PO_4_. Low-N media substituted 150 μM NH_4_NO_3_ (3.75% of replete media concentration) and low-P media substituted 25 μM KH_2_PO_4_ (1.67% of replete media concentration). Solution pH was adjusted to 5.6 with 0.1 N NaOH in each case. Each vertical plate (150 × 15 mm) contained 100 ml of solidified media with half removed to create a headspace for seedling shoots. Washed autoclaved cellophane membrane (Promega, Gel Drying Film, REF V7131) was placed on the half medium. Seedlings were transferred carefully from the germination jars to the cellophane membrane. Each plate contained 8 seedlings, placed at 15 mm intervals. The plates were incubated at a 75° angle in the growth chamber under conditions noted above.

After 1-week incubation, seedlings were inoculated with the *Pseudomonas* strains. For inoculation into vertical plates, *Pseudomonas* cultures were grown overnight in LB liquid media at 27°C. Bacterial pellets were collected and washed with sterile 10 mM MgSO_4_. Pellets were re-suspended in 0.25-strength Johnson's nutrient solution (1.2 mM NO3, 100 μM P, 0.4 mM NH4, 0.5 mM K, 0.2 mM Ca, 0.1 mM Mg, 0.1 mM SO4, 50.5 μM Cl, 20 μM Fe, 20 μM B, 2 μM Mn and Zn, and 0.5 μM Cu, Na, Co, and Mo. Solution pH was adjusted to 5.6 with 0.1 N HCl; Desai et al., 2014) at an estimated bacterial cell concentration of ~1.0 × 10^9^ (OD 600 nm = 1.0). Each seedling root was inoculated with 10 μl of bacterial suspension (~10^7^ cells) or with 10 μl of sterile Johnson's solution. Seedlings were harvested 35 days following bacterial inoculation for phenotype measurements. As in previously published experimental results (Anith et al., 2004; Rojas-Tapias et al., 2012; Majeed et al., 2015), the biomass of bacteria was not collected at the end of incubation and the presence of bacteria was considered only as an experimental treatment in subsequent analyses.

### Aspen seedling phenotype assays

A total of 16 phenotypic measurements were collected from aspen seedlings at the end of the experiment. These included shoot dry weight (mg), shoot length (mm), number of leaves, leaf chlorophyll (Chl) concentration (μg mg^−1^ FW), chl a/b ratio, shoot anthocyanin concentration (μg mg^−1^ FW), shoot ${\text{NO}}_{3}^{-}$ concentration (mg g^−1^ DW), shoot P concentration (mg g^−1^ DW), root dry weight (mg), root branching (integer value), root length (cm), number of rootlets, root anthocyanin concentration (μg mg^−1^ FW), root total N (%), root ${\text{NO}}_{3}^{-}$ concentration (μg g^−1^ DW), and root P concentration (mg g^−1^ DW). Experiments were harvested after 35 days of co-culture. All plates were visually inspected for bacterial or fungal colonies and contaminated plates were discarded. Plant phenotypes corresponding to physical parameters such as shoot length, number of leaves, root length, root branching, and number of rootlets were measured on all seedlings in at least 10 replicate plates per treatment.

Phenotypes that entailed destruction of the samples, such as dry weight (shoot and root), leaf chl, chl a/b ratio, shoot anthocyanin, shoot ${\text{NO}}_{3}^{-}$, shoot P, root anthocyanin, root total percent N, root ${\text{NO}}_{3}^{-}$, and root P were measured on 3 replicate plates per treatment. For dry weight measurements, 4 seedlings were collected from each replicate plate, dried at 65°C for 72 h, and shoot and root tissues separately weighed. Dry tissue was also used for measuring P, and total N concentrations (below). Other biochemical assays (leaf chl, chl a/b ratio, shoot anthocyanin, shoot ${\text{NO}}_{3}^{-}$, shoot P, root anthocyanin, root total percent N, root ${\text{NO}}_{3}^{-}$) were performed using two seedlings per plate per treatment.

Chlorophyll was extracted from whole shoots using cold methanol and quantified according to Porra et al. (1989). Anthocyanins were extracted from root and shoot tissue separately with acidified methanol (1% HCl) and quantified according to Neff and Chory (1998). All assays were adjusted relative to the original protocols to reflect lower biomass amounts and analyzed using a microplate spectrophotometer (Molecular Devices, Sunnyvale, CA, USA).

For analysis of tissue ${\text{NO}}_{3}^{-}$ concentration, dry root or shoot tissue (~10 mg dry weight) was ground and suspended in 300 μl d·H_2_O and kept at 50°C for 1 h. After cooling to room temperature, samples were centrifuged, and 100 μl of supernatant was collected. The nitrate in samples was converted to nitrosalicylic acid by reaction with salicylic acid in concentrated sulfuric acid, and its concentration was then determined spectrophotometrically (Lastra, 2003). Total tissue N was determined on dried root (~5 mg dry weight) by combustion with a Carlo Erba NA 1,500 elemental analyzer (Carlo Erba Strumentazione, Milan, Italy) using acetanilide as a standard. To determine the P concentration of plant root and shoot tissue, dried tissue was ashed at 475°C for 2.5 h, dissolved in 200 μl of 50% concentrated HCl (Sigma ACS reagent), and the crucible rinsed with 800 μl of d·H_2_O and combined with the digest. The resulting 1-ml digests were vortexed until clear, and 100 μl of sample diluted with 100 μl d·H_2_O was used to detect inorganic P using the malachite green method (Martin et al., 1999).

### Analysis of aspen seedling phenotypes

Seedling phenotypes were analyzed using two-factor ANOVA, with the factors being “community” (no bacteria, Pf0-1, Pf-5, SBW25, WH6; 4° of freedom) and “media” (replete, low N, and low P media; 2° of freedom) using “MeV” (v4.5.1, http://www.tm4.org/mev.html). Thresholds for significance were *p* < 0.05, based on 10,000 permutations.

A matrix of Pearson's correlation coefficient (PCC) scores between all pair-wise aspen phenotype measurements across all experimental conditions was generated to identify strong positive and negative correlations between seedling phenotypic measures (95th and 5th percentile of PCC scores, respectively). Strong correlations were visualized as a network (Cytoscape v2.8.0, http://cytoscape.org).

Principal component analysis (PCA) was also performed on seedling phenotypic data. Seedling phenotype were clustered by hierarchical clustering and grouped by PCA using Euclidian distance in R (R v3.0.3). Important principal components (PCs) were considered to be those whose variance exceeded what would be observed if variance were distributed equally across all PCs. Relevant loading values in important PCs from PCA analysis were considered to be those whose absolute value exceeded (1/number of PCs)^0.5^.

### Transportomic modeling

Transportomic capacity of *Pseudomonas* strains was calculated as Predicted Relative Transmembrane Transport (PRTT) scores, as described in Larsen et al. (2015). Briefly all four *Pseudomonas* strains were re-annotated for transporter and sensor functions to insure uniformity of annotations across strains using a custom database of 164,321 protein sequences, annotated with any of 891 KEGG Orthology (KO) annotations (Table S1). Transporter/sensor annotations are associated with one or more of 272 possible ligands. Function was ascribed to a bacterial protein if BLAST-N alignment with >20% sequence similarity and *e* < 1e^−100^. PRTT is a metric that quantifies the relative capacity of a bacterium to transport a ligand across its membrane as a function of the number of genes annotated with transporter functions in its genome. A positive PRTT score indicates an increased relative capacity for transmembrane transport of a specific ligand in the transportome in one bacterium relative to the average transportomic capacity of a set of bacteria. A negative PRTT score indicates a decreased relative capacity for transmembrane transport of a ligand. A PRTT score alone does not necessarily indicate the direction of transport, i.e., import or export, and requires consideration of specific protein annotations. Note that ligand identifications are drawn from the ontology of KEGG compounds and may contain redundant or overlapping terms (e.g., “iron” and “Fe2+”) necessitating additional investigation into the specific transporter proteins or annotations. Table S2 contains the necessary information for readers to make the required associations between ligand, annotation, and predicted *Pseudomonas* protein.

### Correlations between aspen seedling phenotypes and transportomes

Statistically significant correlations between significantly different phenotypes and transportomic models were identified. As the transportomic models are dependent only upon *Pseudomonas* genomic annotation, the matrix of PRTT scores associated with a *Pseudomonas* strain is the same regardless of media condition. Three sets of correlations were considered: Phenotypes significant by community for all media types; phenotypes significant for community-media interaction for only low N stress condition; and phenotypes significant for community-media interaction for only low P stress condition. Statistical significance of the correlations was assigned using a bootstrap approach, randomly re-ordering phenotypes 5,000 times, and determining the frequency at which absolute value of bootstrapped correlations was greater than absolute value of initial correlations. This frequency was expressed as a *p*-value and a significance threshold of *p* ≤ 0.05 was used.

As it is expected that this correlation network will contain false positive interactions, the network was reduced by filtering for edges in the network between nodes that are significantly enriched for specific strain, media condition, or direction of correlations (calculated as a cumulative hypergeometric distribution, *p* < 0.05). The filtered network is comprised of 14 aspen seedling phenotypes and 103 *Pseudomonas* transported ligands, connected by 122 statistically significant correlation edges. All subsequent analysis was performed using this filtered network.

## Results

### Aspen seedling phenotypic analysis

**Figure 1:**
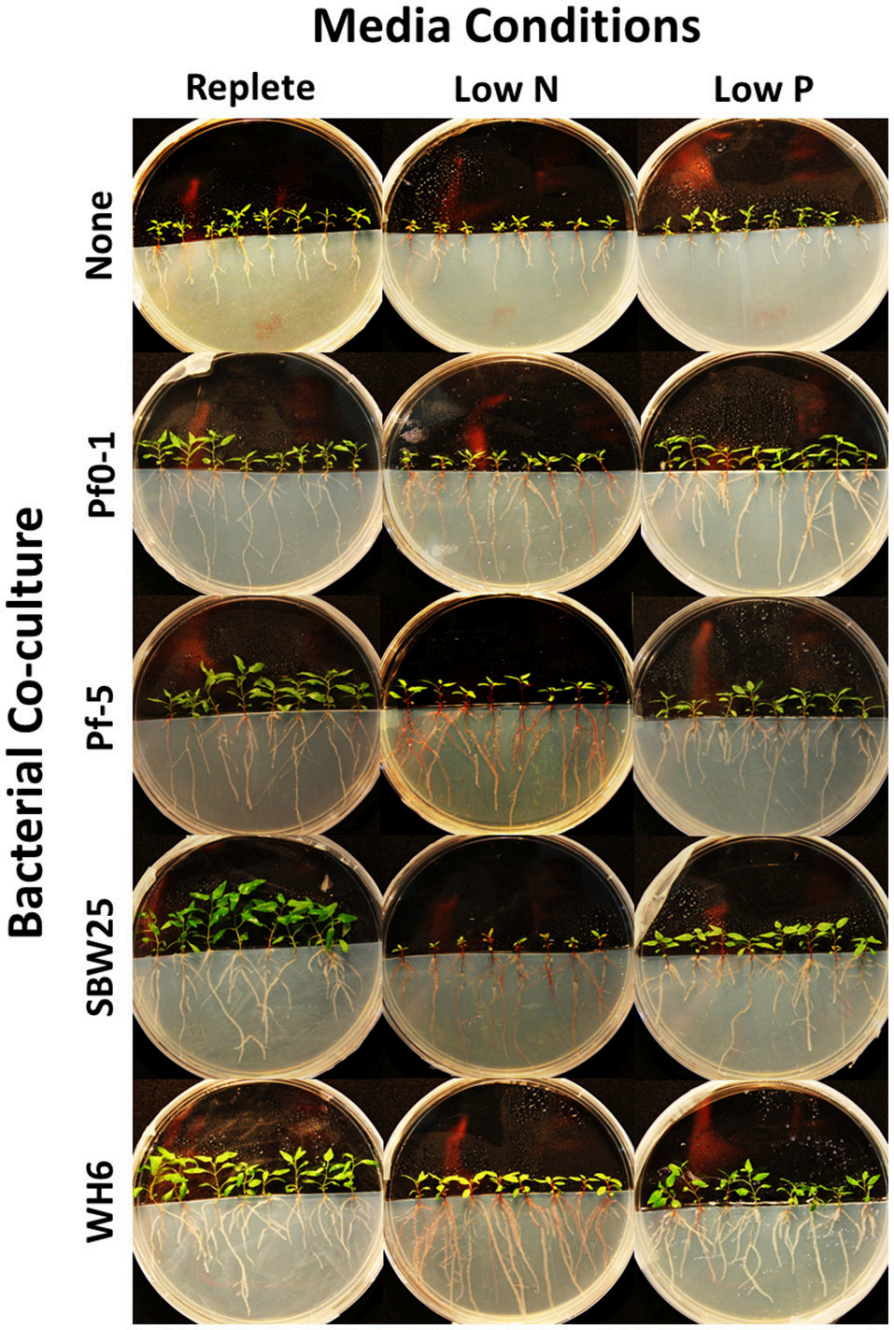
**Impact of PGPB on aspen seedlings under conditions of low nitrogen (N) and phosphorus (P)**. Aspen seedlings inoculated with bacterial strains indicated on the left and grown under replete (4 mM N, 1.5 mM P) or nutrient limiting conditions (low N 150 μM, low P 25 μM) indicated on the top of the image.

Aspen seedling phenotypes were assessed under five “community” conditions (no bacteria, Pf0-1, Pf-5, SBW25, WH6) and three “media” conditions (replete, low N, and low P). A summary of collected quantitative phenotypes can be found in Table 1 and the complete set of collected data can be found in Table S3. Representative seedling vertical plate cultures are pictured in Figure 1. By two-way ANOVA, media composition differentiated 14 (88%) phenotypic characters, 12 (75%) separated by *Pseudomonas* community, and 8 (50%) by the interaction of media and community (Table 1). In aspen seedlings without *Pseudomonas*, N and P limitation led to 52 and 53% reductions in total seedling biomass, respectively (Table 1). Thus, it is clear that nutrient resources limited aspen growth in the delivered low N and P treatments. In addition, the inoculation with four *P. fluorescens* strains resulted in 42% (shoot) and 33% (root) average biomass increases relative to non-bacterial controls in replete nutrient conditions (Table 1), indicating that, in aggregate, the *Pseudomonas* strains confer PGP benefits.

**Table 1:** **Summary of aspen seedling phenotype measurements**.

**Media**	**Replete**					**Low N**					**Low P**					**Media—*p*-value**	**Community—*p*-value**	**Interaction—*p*-value**
**Community**	**None**	**PF-5**	**PF0-1**	**SBW25**	**WH6**	**None**	**PF-5**	**PF0-1**	**SBW25**	**WH6**	**None**	**PF-5**	**PF0-1**	**SBW25**	**WH6**			
Number of leaves	6.37 (0.45)	6.70 (0.62)	6.25 (0.33)	7.75 (0.43)	6.83 (0.36)	5.00 (0.37)	6.25 (0.00)	6.00 (0.37)	5.58 (0.38)	7.00 (0.33)	5.62 (0.12)	6.79 (0.26)	6.12 (0.12)	6.83 (0.31)	6.33 (0.19)	**0.000**	**0.000**	**0.000**
ChlA/ChlB	1.10 (0.11)	1.12 (0.03)	1.14 (0.02)	1.11 (0.06)	1.15 (0.00)	1.56 (0.66)	1.08 (0.11)	1.05 (0.20)	1.09 (0.08)	1.16 (0.04)	0.77 (0.29)	1.10 (0.05)	0.76 (0.42)	1.01 (0.03)	0.81 (0.00)	**0.000**	0.698	0.112
Chlorophyll	1.12 (0.06)	1.37 (0.91)	1.36 (0.25)	1.66 (1.20)	1.10 (0.01)	0.56 (0.05)	1.00 (0.49)	0.59 (0.31)	0.51 (0.21)	1.15 (0.66)	0.41 (0.22)	1.17 (0.67)	0.68 (0.35)	0.79 (0.34)	1.29 (0.00)	**0.010**	0.236	0.609
Shoot NO_3_ (μg/g)	31.17 (7.49)	42.68 (17.54)	44.82 (1.65)	42.01 (5.48)	56.64 (12.55)	36.07 (17.21)	11.90 (3.71)	13.25 (4.21)	12.24 (1.70)	27.19 (24.46)	45.37 (12.53)	45.61 (8.50)	63.26 (8.87)	24.38 (7.78)	55.00 (44.40)	**0.000**	**0.049**	0.097
Shoot anthocyanin	0.33 (0.21)	0.47 (0.00)	0.46 (0.04)	0.41 (0.10)	0.44 (0.06)	0.53 (0.00)	0.61 (0.04)	0.57 (0.17)	0.74 (0.04)	0.44 (0.02)	0.52 (0.05)	0.54 (0.05)	0.52 (0.00)	0.97 (0.58)	1.68 (1.00)	**0.001**	**0.043**	**0.010**
Shoot P (μg/g)	1.46 (0.47)	1.67 (0.43)	1.59 (0.60)	1.09 (0.59)	0.94 (0.16)	0.99 (0.10)	1.96 (0.76)	2.61 (1.25)	0.77 (0.11)	0.80 (0.22)	0.68 (0.14)	1.30 (0.68)	2.08 (1.52)	0.53 (0.13)	0.92 (0.00)	0.366	**0.001**	0.592
Shoot length (cm)	1.20 (0.06)	1.43 (0.08)	1.23 (0.20)	1.87 (0.11)	1.45 (0.05)	0.58 (0.10)	1.20 (0.01)	0.91 (0.10)	0.90 (0.06)	1.18 (0.14)	1.01 (0.05)	1.15 (0.18)	1.02 (0.13)	1.31 (0.13)	1.27 (0.22)	**0.000**	**0.000**	**0.000**
Shoot dry weight (g)	6.83 (1.20)	11.13 (0.66)	10.06 (1.34)	13.16 (0.70)	14.26 (4.02)	2.63 (0.23)	9.00 (1.90)	8.76 (1.33)	6.21 (3.75)	10.66 (1.13)	3.86 (1.49)	8.90 (1.25)	10.00 (1.15)	12.33 (1.55)	13.06 (0.76)	**0.000**	**0.000**	0.118
Root NO_3_ (μg/g)	36.64 (21.11)	48.58 (1.18)	49.70 (14.48)	33.05 (6.36)	37.58 (7.02)	29.78 (19.76)	16.19 (9.05)	19.69 (7.18)	11.29 (1.51)	22.30 (12.77)	35.07 (24.77)	53.84 (31.07)	45.50 (2.24)	28.64 (3.68)	88.42 (NA)	**0.000**	**0.016**	**0.014**
Root %N	3.54 (1.01)	3.06 (0.68)	2.76 (0.45)	2.86 (0.32)	2.62 (0.04)	0.50 (0.00)	2.97 (0.84)	2.14 (0.15)	1.92 (0.27)	1.40 (0.00)	2.00 (0.04)	2.71 (0.36)	2.48 (0.17)	1.96 (0.12)	3.78 (0.17)	**0.000**	**0.001**	**0.000**
Root anthocyanin	0.07 (0.02)	0.21 (0.23)	0.13 (0.07)	0.21 (0.01)	0.18 (0.14)	0.51 (0.01)	0.27 (0.09)	0.56 (0.28)	0.63 (0.02)	0.57 (0.60)	0.38 (0.11)	0.20 (0.08)	0.22 (0.06)	0.55 (0.56)	1.25 (1.00)	**0.008**	0.079	0.192
Root P(mg/g)	2.21 (0.18)	3.32 (0.88)	4.04 (1.88)	1.90 (0.62)	1.75 (0.40)	0.93 (0.17)	1.88 (0.27)	2.53 (0.60)	0.78 (0.20)	2.82 (2.75)	0.59 (0.26)	0.90 (0.29)	1.23 (0.70)	0.40 (0.09)	1.32 (0.00)	**0.000**	**0.009**	0.290
Root branching	2.65 (0.28)	2.95 (1.25)	2.65 (0.20)	3.83 (0.19)	1.83 (0.93)	3.13 (0.37)	3.75 (1.47)	4.83 (0.68)	4.95 (2.06)	2.41 (0.85)	1.30 (0.33)	3.29 (1.06)	3.12 (0.12)	5.58 (1.60)	2.37 (0.25)	**0.023**	**0.000**	0.196
Rootlets	2.45 (0.68)	3.87 (0.33)	3.45 (0.10)	4.50 (0.69)	3.75 (0.33)	3.18 (0.42)	4.08 (0.68)	3.45 (0.43)	3.83 (0.83)	5.00 (0.76)	2.62 (0.45)	3.33 (0.14)	3.58 (0.14)	4.83 (0.38)	3.83 (0.31)	0.212	**0.000**	**0.025**
Root length (cm)	2.80 (0.86)	3.74 (1.07)	2.99 (1.31)	4.00 (0.23)	2.11 (0.00)	6.05 (1.79)	4.63 (0.68)	5.48 (0.64)	5.03 (0.52)	4.77 (0.59)	1.62 (0.30)	3.77 (0.35)	2.83 (0.63)	2.82 (0.43)	2.52 (0.42)	**0.000**	0.115	**0.026**
Root dry weight (g)	2.10 (0.62)	2.33 (0.51)	2.70 (0.17)	3.60 (0.78)	3.36 (0.80)	1.63 (0.25)	4.60 (1.24)	4.86 (0.63)	3.56 (0.35)	4.96 (0.90)	0.80 (0.09)	3.66 (0.35)	3.06 (0.83)	4.73 (0.37)	1.90 (0.26)	**0.000**	**0.000**	**0.000**

*All values are presented as an average (standard deviation) of biological replicates. “Replete,” “Low N,” and “Low P” refer to media condition. “None,” “Pf-5,” “Pf0-1,” “SBW25,” and “WH6” refer to community composition. Statistical analysis is reported from two-way ANOVA. P < 0.05 are highlighted (bold)*.

#### Low N and P conditions alter pigments in aspen seedlings

Three phenotypic measures were solely influenced by media treatment (total chlorophyll, chlorophyll a/b, root anthocyanin; Table 1). Considering average values across the 5 community treatments, total chlorophyll in seedlings grown with low N was 57% of that in seedlings grown with replete media but the chlorophyll a/b ratio did not change. In contrast, total chlorophyll in low P derived seedlings was intermediate between replete and low N plants, yet the chlorophyll a/b ratio was ~75% of that in the other media treatments (Table 1). Root anthocyanin concentration was strongly stimulated by both N limitation (3.1-fold) and P limitation (3.2-fold) across all treatments. This effect was particularly strong in non-bacterial aspen seedlings where anthocyanin concentration increased 7.2-fold in low N and 5.4-fold in low P relative to replete condition (Table 1). However, in replete media the presence of *Pseudomonas* strains increased 2- to 3-fold root anthocyanin accumulation (Table 1). These observations suggest that anthocyanin accumulation in aspen seedling roots can be induced by multiple environmental cues including nutrient limitation and the presence of bacteria.

#### *Pseudomonas* affects P acquisition in aspen

Shoot P was the only phenotype solely influenced *Pseudomonas* community (Table 1). Across media conditions, seedlings co-cultured with Pf-5 and Pf0-1 exhibited 1.6- and 2-fold greater shoot P content relative to seedlings without bacteria, respectively; SBW25 and WH6 did not differ from non-bacterial aspen (Table 1). Root P accumulation decreased 3.7-fold in non-bacterial seedlings grown with low P relative to replete media. Co-culture of seedlings with all *Pseudomonas* strains elevated root P concentration relative to non-bacterial seedlings, with strongest effect for strains WH6 (2.1-fold) and Pf0-1 (2.2-fold). Taken in aggregate, total P accumulation (μg plant^−1^, calculated as Root DW × Root P + Shoot DW × Shoot P) was significantly greater in all *Pseudomonas*-colonized plants compared to non-bacterial plants and exhibited strain-specific differences in the order Pf0-1 (28.9) = Pf-5 (22.5) = WH6 (18.8) > SBW25 (12.4) > non-bacterial control (7.3).

#### *Pseudomonas* co-culture and media have a combinatorial effect on aspen seedling phenotypes

**Figure 2:**
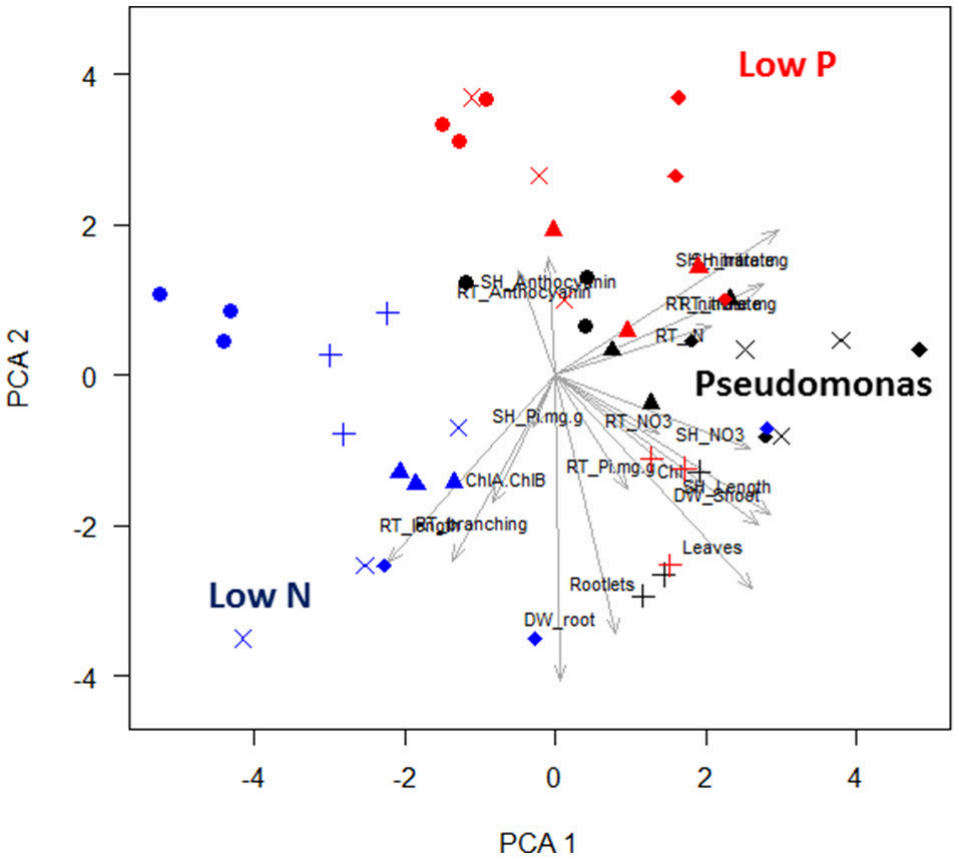
**Principal component analysis and hierarchical clustering of aspen seedling phenotypes**. A scatter plot of the first two PC is shown. Each point is a single observation, drawn from average values of individual seedlings on a vertical plate. Shape indicates culture condition: no bacteria are circles, Pf-5 are triangles, Pf0-1 are crosses, SBW25 are × 's, and WH6 are diamonds. Nutrient condition is indicated by color: replete media is black, low N media is blue, and low P media is red. In PCA plot, blue, red, and black labels have been added to highlight the effects of colonization by bacteria and the effects of nutrient stress. Labeled arrows are the eigenvectors associated with seedling phenotype measure.

Most aspen phenotypes responded to combinations of media and *Pseudomonas* community or their interaction (Table 1), indicating that PGP bacteria mediate aspen response to abiotic resource availability. PCA of seedling phenotypic data identified five PCs that together accounted for 80% of the total variance (Figure S1). The first two PCs, which account for 43% of the variance (Table S4), can be linked to specific culture conditions. The first PC includes six phenotypic characters (leaf chlorophyll concentration, leaf count, shoot length, shoot dry weight, root %N, and root length; Figure 2), highlighting shoot-root tradeoffs as aspen seedlings increased root elongation as a N acquisition strategy. The second PC also includes root growth relationships and seedling N nutrition, specifically feedbacks between tissue ${\text{NO}}_{3}^{-}$ accumulation and root architecture (Figure 2). PC3 separates anthocyanin responses and root P, suggesting this PC encompasses aspen stress-response to P acquisition. Graphically, PCA clustering of data indicates a distinct grouping of data, with low N, low P, and presence of *Pseudomonas* each having distinctive effects on aspen seedling phenotypes (Figure 2).

#### Pseudomonads provide strain-specific benefits to aspen seedlings

Patterns of aspen seedling shoot and root architecture and biomass (Table 1) indicate that Pseudomonads generally increase C allocation to roots under both N and P limitation relative to non-bacterial seedlings, and these changes, while variable among strains, may underpin PGP effects. Strain SBW25 promoted an increase in several seedling shoot phenotypes (chlorophyll, number of leaves, shoot length, shoot DW) in replete media condition. However, these positive effects were not observed under nutrient limitation where seedlings co-cultured with SBW25 performed similarly to non-bacterial seedlings. Under N limiting conditions, root length increased markedly (2.1-fold) and root mass decreased slightly (0.76-fold) in non-bacterial seedlings relative to replete conditions. For seedlings grown in low N media and inoculated with Pf0-1, WH6, and Pf-5, and to a lesser extent with SBW25, root mass was increased (up to 3-fold) and root length declined (up to 0.76-fold) relative to non-bacterial seedlings. Low N also reduced shoot length (0.5-fold) and shoot mass (0.38-fold) in non-bacterial seedlings relative to replete conditions. Under N limitation, Pf-5, Pf0-1, and WH6 restored shoot length and shoot mass to levels greater than that of the non-bacterial control in replete media.

Under P limitation, root length and root mass of non-bacterial seedlings decreased 0.57- and 0.38-fold, respectively, relative to replete conditions. Strain Pf-5 was the most efficient at increasing root length (2.3-fold) and root mass (4.5-fold) under low P compared to non-bacterial treatments. Strains Pf0-1, SBW25, and WH6 also had positive effects, restoring root length and mass above the non-bacterial control with replete media (Table 1). Low P also reduced shoot mass (0.57-fold) and shoot length (0.84-fold) in non-bacterial seedlings relative to replete conditions. Strain WH6 maintained shoot mass under low P to levels similar to that observed for *Pseudomonas*-treated seedlings in replete media. Of note, these strains did not affect shoot length under P limitation. Together, these observations suggest that *Pseudomonas* treatments increase resource allocation to root and shoot in a strain-specific manner under N and P limitation. These PGP effects and alleviation of stress likely result from multiple and strain-specific PGP mechanisms functioning under different resource environments.

### *Pseudomonas* strains have unique transportomic capacities

**Figure 3:**
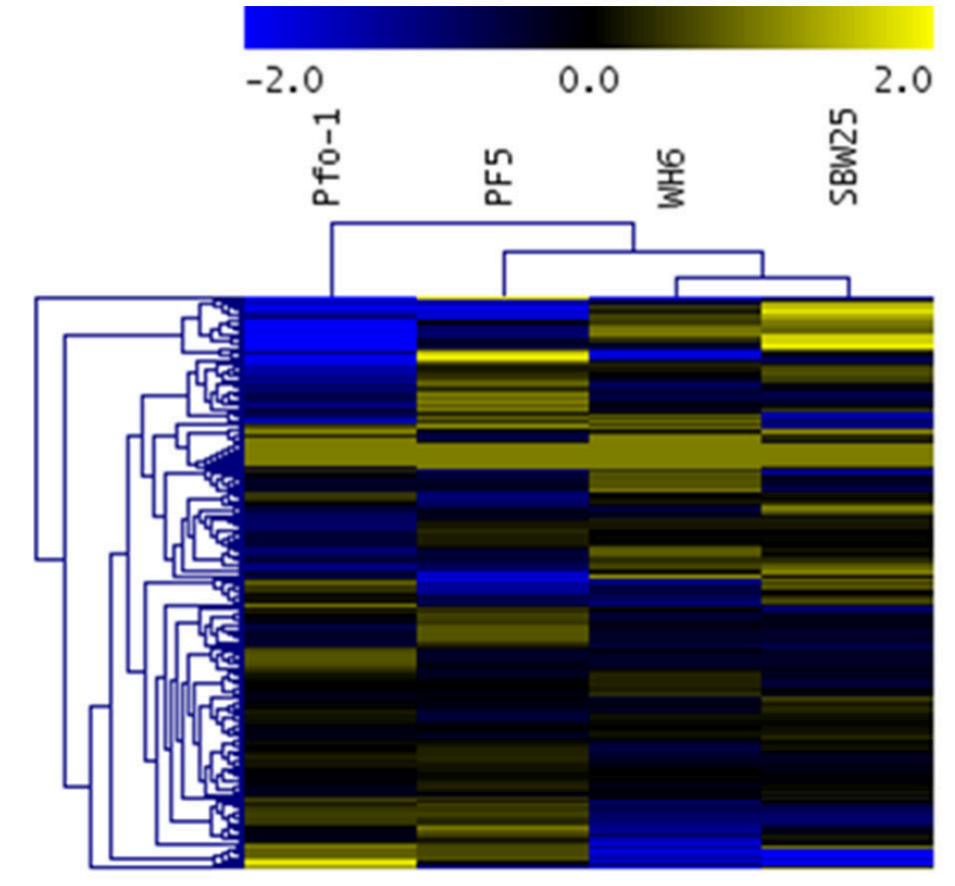
**Heat map and hierarchical cluster of ***Pseudomonas*** strain PRTT-scores**. In this heat map, rows are *Pseudomonas* strains and columns are specific ligands. Values in heat map are PRTT-scores.

While statistically significant differences in aspen phenotypes suggest *Pseudomonas* strain-specific beneficial effects that depend on nutrient availability, the statistical analysis does not provide information on the molecular mechanisms underpinning these beneficial effects. The relative transportomic capacity of *Pseudomonas* strains Pf-5, Pf0-1, SBW25, and WH6 were quantified using PRTT-scores. There are 199 ligands in the calculated *Pseudomonas* transportome, and the complete set of PRTT-scores can be found in Table S4 and is summarized as a hierarchical cluster and heat map in Figure 3.

Different *Pseudomonas* strains can be distinguished by their unique profile of strongest transportomic capacity, defined here as having a PRTT-score for a specific ligand that is at least 2-fold greater than the average PRTT score for the other strains.

Pf0-1: Mangenese, lipopolysaccharide, putrescine, microcin, iron, Fe^2+^, Fe^3+^, chloramphenicol, hemolysin, mineral and organic ion transporters, and spermidine.
Pf-5: Pores ion channels, L-arginine, L-ornithine, zinc cation, sorbitol, glycine, trimethylamine-n-oxide, n-acetylglucosamine, tungstate, lactose, alpha-glucoside, teichoic acid, sulfate, tetracycline, maltodextrin, l-threonine, and SN-glycerol 3-phosphate.
SBW25: L-glutamine, nickel, nickel^2+^, L-leucine, glucose, L-arabinose, peptide, ribose, sodium cation, cytosine, thymine, electrochemical potential-driven transporters, spermidine, putrescine, uracil, L-isoeucine, L-valine, dimethyl sulfoxide, fructose, multidrug transporters, xylose, l-aspartate, l-glutamate, adenine, nucleoside, formate, ascorbate, and oxalate.
WH6: Cobamide coenzyme, peptide, ABC-2 type and other transporters, fructose, L-aspartate, L-glutamate, nitrate, nickel, nickel^2+^, ribose, mannose, L-proline, unknown transporters, gluconate, lipoprotein, cytosine, and thymine.

With 29 ligands, WH6 has the most unique transportomic capacities relative to the other strains.

From the hierarchical cluster (HCL) figure of PRTT-scores (Figure 3), the transportomic capacity of Pf-5 and Pf0-1 are more similar to one another than the other strains and WH6 and SBW25 are more similar to one another than the others. The transportomic capacities that distinguish Pf-5 and Pf0-1 from WH6 and SBW25 (by *t*-test, *p* < 0.05) are for the ligands galactarate, 3-hydroxyphenylpropionic, trehalose, C4-dicarboxylate, lactate, drug transporters, cadaverine, D-allose, L-phenylalanine, L-tyrosine, dehydroshikimate, shikimate, and iron.

### Aspen seedling phenotype and *Pseudomonas* transportome correlation networks

In order to identify potential mechanisms by which the observed *Pseudomonas* strain-specific differences in aspen seedling phenotypes might be linked to differential transportomic capacities, we considered the correlation network generated between observed aspen seedling phenotype measurements and predicted *Pseudomonas* transportomes.

#### Aspen seedling phenotypes are not independent of one another

**Figure 4:**
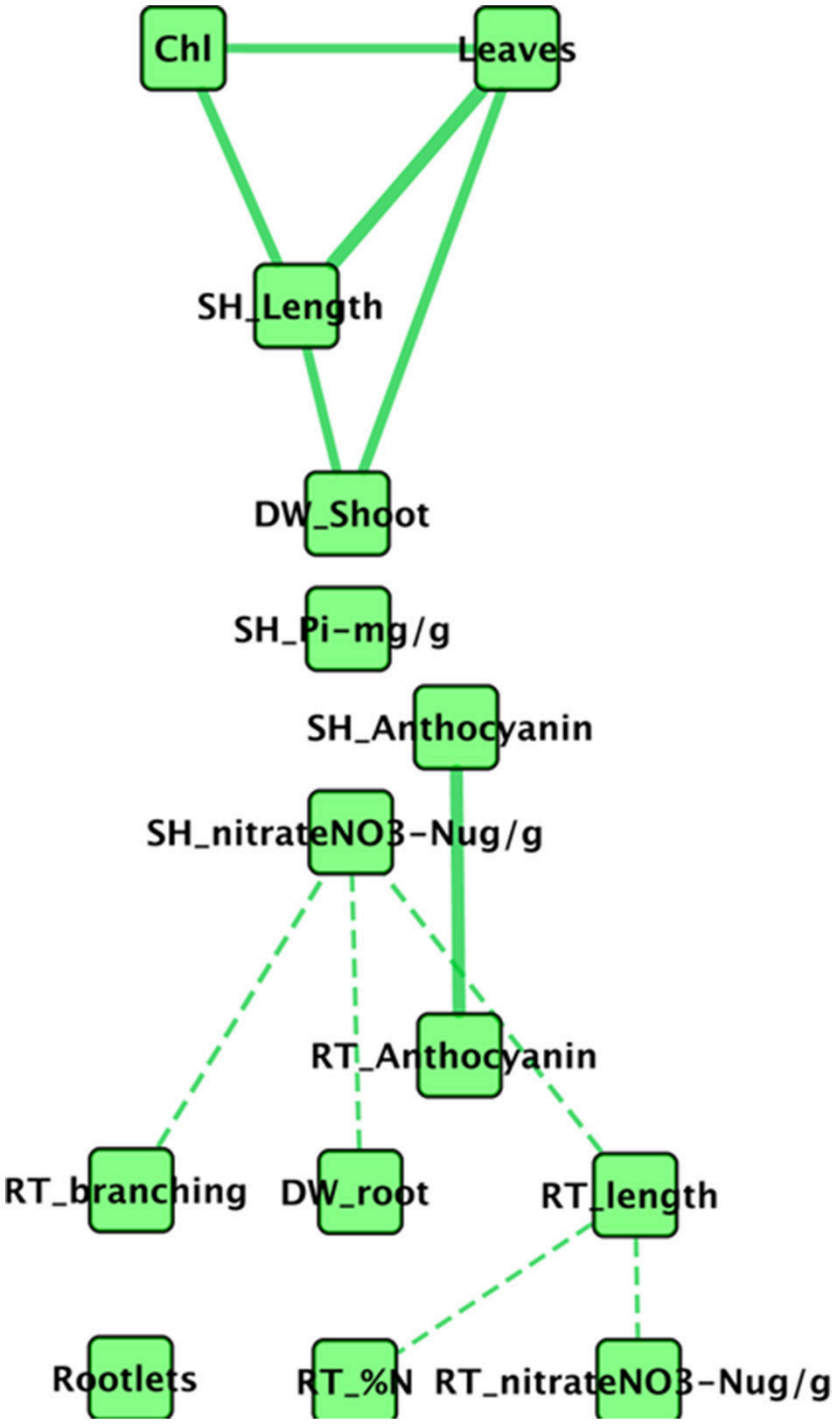
**Network of correlated aspen seedling phenotypes**. In this network, nodes are measured seedling phenotypes, solid edges indicate strong positive correlations, and dashed edges indicate strong negative correlations. The phenotypes for root and shoot P concentration do not correlate strongly with any phenotypes.

Intuitively, it seems likely that all aspen seedling phenotypes are not independent of one another. For example, root length and root dry weight might reasonably be expected to correlate, as might be number of leaves and above ground shoot dry weight. To better understand how *Pseudomonas* transportomic capacity might be linked to aspen seedling phenotypes, we first considered how those seedling phenotypes might be interdependent on one another by constructing a correlation network between phenotypic measurements (Figure 4). This network is comprised of 16 nodes and 11 edges. Chlorophyll a/b, shoot P, root N, and rootlets do not appear in the set of significantly correlated phenotypes. The network is comprised of three connected subnetworks: mostly belowground phenotypes, aboveground phenotypes, and anthocyanins. In the aboveground network, shoot dry weight, leaf count, stem length, and chlorophyll concentrations are positively correlated. Root and shoot anthocyanins are positively correlated. The belowground subnetwork is comprised entirely of negative correlations between root phenotypes and between root architecture (branching, length, and dry weight) and shoot ${\text{NO}}_{3}^{-}$ concentration.

#### Correlations networks between aspen seedling phenotypes and *Pseudomonas* transportomes predict molecular mechanisms of PGP

Possible mechanisms linking changes in seedling phenotype to relative, differential transportomic capacity of co-cultured *Pseudomonas* strains were sought by identifying significant correlations between transportomic capacity (expressed as PRTT-scores, and available in Table S5) and phenotypes (Table S2). The initial network (Table S6) is comprised of a completely connected network with 143 *Pseudomonas*-transported ligands, 15 aspen seedling phenotypes, and 219 statistically significant (*p* < 0.05) correlations between ligands and phenotypes. Edges are categorized as positively or negatively correlated, with the majority (80%) of statistically significant edges being positive correlations. Ligands were categorized by the *Pseudomonas* strain with the highest PRTT-score for that ligand. Highest PRTT-score ligands were fairly evenly distributed between Pf0-1 (21%), Pf-5 (23%), WH6 (26%), and SBW25 (30%).

**Figure 5:**
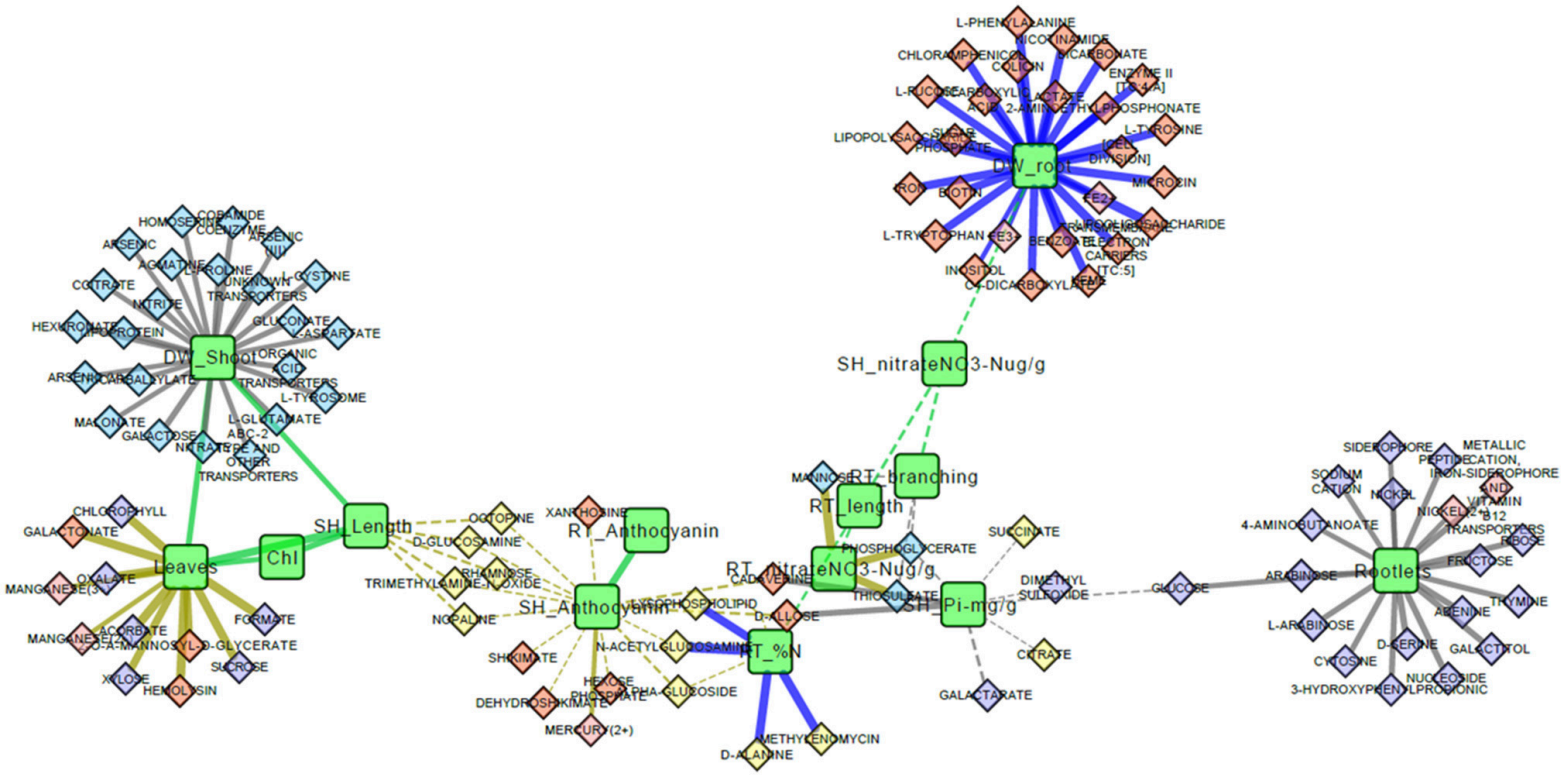
**Phenotype and ***Pseudomonas*** transportome correlation network**. This is a graphical representation of the correlation network for aspen phenotypes and *Pseudomonas* transportome. Green rounded rectangles are aspen seedling phenotypes and diamonds are predicted ligands transported by *Pseudomonas* strains. Ligands are colored according to the specific *Pseudomonas* strain with the greatest (by PRTT-score) relative capacity for that ligand's transport: yellow for Pf-5, orange for Pf0-1, red for SBW25, and purple for WH6. Wavy green lines are strong correlations between aspen seedling phenotypic features. Solid straight lines are strong correlations between *Pseudomonas* transportomic capacity and aspen seedling phenotypes colored by culture condition: gray for replete media, blue for low N media, and yellow for low P media.

The complete correlation network was refined to include only enriched interaction classes (Table 2) and the resulting network was comprised of 14 phenotypes and 103 *Pseudomonas* transportomic functions, linked by 131 interactions (Figure 5). Predictions of molecular mechanisms of PGP in subsequent Discussion Section utilize this, refined network in analyses.

**Table 2:** **Enrichment of interactions in Transportome-Phenotype interaction network**.

	**Root Dry Weight (57)**	**Root Branching (4)**	**Rootlets (31)**	**Root %N (9)**	**Root NO3 (6)**	**Shoot Dry Weight (56)**	**Shoot Length (14)**	**Shoot Anthocyanin (17)**	**Shoot P (9)**	**Number of Leaves (15)**
Pfo-1 (46)	**8.36E-08**	6.13E-01	9.31E-01	8.85E-01	6.96E-01	1.00E+00	5.95E-01	**1.13E-02**	2.87E-01	6.78E-02
Pf-5 (51)	2.07E-01	2.32E-01	9.98E-01	**4.07E-05**	8.39E-02	9.78E-01	**1.02E-04**	**5.53E-03**	3.51E-01	9.84E-01
WH6 (56)	3.68E-01	**3.94E-03**	9.96E-01	4.16E-01	**1.59E-02**	**2.98E-04**	9.86E-01	8.59E-01	4.16E-01	3.29E-01
SBW25 (66)	1.00E+00	7.65E-01	**2.43E-11**	9.63E-01	8.37E-01	**3.13E-03**	6.56E-01	9.98E-01	2.70E-01	1.26E-01
Low N (68)	**0.00E**+**00**	7.77E-01	1.00E+00	**2.73E-02**	8.47E-01	1.00E+00	4.50E-01	9.99E-01	9.67E-01	5.23E-01
Low P (56)	1.00E+00	6.96E-01	7.31E-01	**4.98E-02**	**0.00E**+**00**	1.00E+00	**2.58E-05**	**0.00E**+**00**	9.34E-01	**7.04E-06**
All Media (95)	1.00E+00	**0.00E**+**00**	**6.10E-06**	9.95E-01	9.44E-01	**0.00E**+**00**	1.00E+00	1.00E+00	**0.00E**+**00**	1.00E+00
Negative (44)	1.00E+00	**0.00E**+**00**	9.12E-01	2.62E-01	5.65E-02	1.00E+00	**1.71E-03**	**8.08E-12**	**1.17E-05**	9.70E-01
Positive (175)	**6.28E-06**	9.99E-01	**2.73E-02**	4.29E-01	7.36E-01	**2.59E-06**	9.91E-01	1.00E+00	1.00E+00	**0.00E**+**00**

*Enrichment of predicted interactions for specific phenotype-transportome categories are indicated. Transportomic categories are Pseudomonas strain (Pf0-1, Pf-5, WH6, or SBW25) with strongest capacity to transport specific ligand (i.e., highest PRTT-score); media condition (replete, low N, or low P); and direction of correlation (positively or negatively correlated). Numbers in parentheses indicate total number interactions in correlation network that belongs to category. P-values were calculated as Cumulative Hypergeometric Distribution and highlighted (bold) for values < 0.05*.

## Discussion

Interactions between aspen seedlings and *Pseudomonas* strains under varying nutrient environments provide insight into how PGP benefits of this rhizobacteria could be linked to the annotated function of transmembrane transporter proteins. Key observations include: (i) low N and low P induce stress in non-bacterial seedlings under these experimental conditions; (ii) *Pseudomonas* can ameliorate nutrient limitation in strain and media-specific fashions; (iii) *Pseudomonas* strains have unique transportomic capacities; and (iv) there are significant correlations between aspen seedling phenotype and *Pseudomonas* transportomes. From these analyses, it becomes possible to hypothesize the molecular mechanisms underlying strain and media-specific effects of *Pseudomonas*-aspen PGP interactions.

### Analysis of phenotype-transportome correlation network identifies potential PGP mechanisms

There are two principle mechanisms by which *Pseudomonas* provides PGP effects to aspen seedlings that can be inferred from analysis of the correlations between *Pseudomonas* transportomic capacity and seedling phenotypes. Pseudomonads may: (i) increase the availability of nutrients to the seedlings; and/or (ii) influence root architecture through release of regulatory compounds.

#### *Pseudomonas* increase nutrient acquisition to aspen seedlings

Organic acids (e.g., citrate, malate, and oxalate) exuded by roots mobilize mineral nutrients for uptake by plant roots (Jones and Darrah, 1994). These benefits are best expressed when exudates alter mineral solubility equilibria in the rhizosphere. The balance between organic acid transport and utilization by rhizobacteria may similarly contribute to nutrient dissolution and promote both root and shoot growth by increasing nutrient availability. In the current study, the transport systems of the *Pseudomonas* strains exporting organic acids and other siderophores are predicted to contribute PGP benefits. However, Pf0-1, which had the highest PRTT score for organic acid transport, did not confer a growth advantage to aspen seedlings relative to other strains, although tissue P concentrations were high (Table 1). In our experimental system, bacterial release of nutrients may not drive seedling response because the nutrients are all provided in inorganic soluble forms and are presumably available to the plant. The availability of other ions not measured in aspen, such as Fe^2/3+^, may be altered by siderophores released by *Pseudomonas* and may have benefited aspen growth (Tian et al., 2009; Shen et al., 2012).

#### *Pseudomonas* influences aspen seedling root architecture

Pseudomonads had a significant effect on aspen root architecture (root branching, rootlets) and shoot biomass independent of media condition. The strongest effects on *Pseudomonas* root architecture were by SBW25 for root branching (*p*-value 3.9 × 10^−3^) and WH6 for rootlets (*p*-value 2.4 × 10^−11^), and these effects may have contributed to the shoot biomass gains in both SBW25 (*p*-value 3.0 × 10^−4^) and WH6 (*p*-value 3.1 × 10^−3^; Table 1). Changes in root architecture may have two consequences to PGP: increasing root branching and rootlets promotes nutrient uptake by increasing the root surface area for absorption and may also provide a more favorable environment for *Pseudomonas* growth.

Molecules with potential transporters in the *Pseudomonas* transportomes that have been shown to influence root morphology include several of the sugars (Baskin et al., 2001; Stevenson and Harrington, 2009), polyamines (Couee et al., 2004), and glutamate (Walch-Liu and Forde, 2007). These molecules and related compounds could be produced and released by *Pseudomonas* or alternatively produced by the plant and taken up and utilized by *Pseudomonas*. Both of these processes may take place simultaneously into the rhizosphere of aspen and play roles in increasing root growth and surface area. *Pseudomonas* may take up and utilize a variety of photosynthetically-derived sugars commonly found in the root exudates (e.g., glucose, xylose, galactose, and fructose; Walker et al., 2003; Carvalhais et al., 2013) enhancing its ability to grow and survive in the rhizosphere. *Pseudomonas* may additionally produce auxin (Larsen et al., 2015) or alter auxin levels/signaling pathways in the host root (Walch-Liu and Forde, 2007), which would alter patterns of lateral root development (Salazar-Henao et al., 2016). From analysis of interaction network (Figure 5), root biomass is also predicted to be influenced by the transport of the possible regulatory compounds tryptophan, phenylalanine, nicotinamide, and lipopolysaccharides by *Pseudomonas*. Tryptophan is a known plant growth promoter that interacts with root-associated bacteria (Hassan and Bano, 2015). Tryptophan is also a precursor to auxin biosynthesis (Zhao, 2012) and may mediate root architecture change through this pathway. Phenylalanine induces secondary metabolism in plants (Basha et al., 2006; Koca and Karaman, 2015). Nicotinamide is a plant stress-associated compound that can induce and regulate secondary metabolic accumulation and induce plant defense responses (Takeuchi et al., 1975; Mohamed et al., 1989; Hashida et al., 2007). Interestingly, several of the sugars for which there are transporters in *Pseudomonas* have complex effects on anthocyanin accumulation in *Arabidopsis* when supplemented in the media (Stevenson and Harrington, 2009). In our study the observed increase of anthocyanin concentrations in replete media (presumed non-stressed) aspen seedling co-cultured with *Pseudomonas* may reflect the plant response to the differential metabolism of some of these sugars by *Pseudomonas*.

In our vertical plate system, increased root growth and surface area should increase aspen seedlings access to nutrients. In the media, both ${\text{NO}}_{3}^{-}$ and H_2_${\text{PO}}_{4}^{-}$ are soluble and limitations to their diffusion in agar are expected to be small. Nonetheless, while phenotypes such as tissue concentrations of ${\text{NO}}_{3}^{-}$ and P did not reflect beneficial effect of *Pseudomonas* (Table 1), total P accumulation in aspen seedlings co-cultured with *Pseudomonas* increased by 1.7- to 3.9-fold and final seedling biomass was correlated to rootlet formation. It thus appears that one of the PGP effects of *Pseudomonas* is enhanced nutrient acquisition related to altered root architecture, as reported previously (Frey-Klett et al., 2011).

#### *Pseudomonas* enhances colonization of the rhizosphere

Some compounds transported by *Pseudomonas* may directly stimulate or repress the defense response of aspen seedling roots. Markers of pathogenicity (octopone, nopaline, glucosamine, and rhamnose) are all negatively correlated with shoot length in low P conditions (Figure 5). Rhamnose (Santhanam et al., 2016) and nopaline and octopine (Lippincott and Lippincott, 1970) are pathogenicity-related markers associated with root crown galls. Glucosamine is a component of chitin that may induce plant defense responses (Nurnberger and Brunner, 2002) and glucosame is also a nitrogenous compound known to be rapidly taken up by plant root (Roberts, 1970). Proline, predicted to influence shoot biomass, has been observed to induce stress tolerance in basil (Rady et al., 2016).

Other compounds that may be transported by systems coded in the *Pseudomonas* transportome may mediate positive interactions and/or facilitate colonization between bacteria and roots. Transport of lipoproteins, lipopolysaccharides, and fucose are all correlated with seedling biomass (Figure 5). Lipoproteins are essential to forming biofilms and colonizing plant roots (Campisano et al., 2006; Ghafoor et al., 2011) and lipopolysaccharides are known mediators between plant roots and soil bacteria (Duijff et al., 1997; Reitz et al., 2000; Fedonenko et al., 2001). Lipopeptides are regulatory compounds in *Pseudomonas*-root interactions that influence antimicrobial activity, motility, and biofilm formation (Song et al., 2014). Fucose is a component of plant root mucilage (Roy et al., 2002) and is known to promote interactions with rhizosphere community (Northcote and Gould, 1989).

### Conclusions and future studies

We sought to uncover potential molecular mechanisms underlying rhizobacterial plant growth promotion effects under conditions of limited N and P using a laboratory model of aspen seedlings and four strains of *Pseudomonas*, Pf-5, Pf0-1, SBW25, and WH6. We demonstrated that our nutrient limited conditions produced measurable stress responses in aspen seedlings and that those responses were alleviated by *Pseudomonas* in a strain and media-specific fashion. We propose that the alleviation of nutrient stress by *Pseudomonas* is due to three classes of ability functions: to mobilize nutrients, to direct aspen seedling root structure, and to successfully colonize the rhizosphere. These mechanisms are potentially linked to the transport of specific ligands, which can be traced back to specific genes and proteins for transporters and sensors in *Pseudomonas*. These results will lead directly to future specific, hypothesis-driven biological experiments to validate predicted PGP transportomic mechanisms.

## Author contributions

All authors contributed to experimental design. SS and JC oversaw biological experiments and analysis. PL performed computational analysis and all authors contributed to analysis of results. All authors have read and approved the final manuscript.

## Funding

This contribution originates in part from the “Environment Sensing and Response” Scientific Focus Area (SFA) program at Argonne National Laboratory. The submitted manuscript has been created by UChicago Argonne, LLC, Operator of Argonne National Laboratory (“Argonne”). Argonne, a U.S. Department of Energy Office of Science laboratory, is operated under Contract No. DE-AC02-06CH11357. The U.S. Government retains for itself, and others acting on its behalf, a paid-up non-exclusive, irrevocable worldwide license in said article to reproduce, prepare derivative works, distribute copies to the public, and perform publicly and display publicly, by or on behalf of the Government.

## Availability of supporting data

All experimental and model data are available as Supplemental Data. Perl code for calculation of PRTT-scores is available upon request.

### Conflict of interest statement

The authors declare that the research was conducted in the absence of any commercial or financial relationships that could be construed as a potential conflict of interest.

## Supplementary material

Figure S1 **Bar graphs of amount of variance each PC contributes to PCA**.

Table S1 **Transporter function profiles for re-annotated Pseudomonads Pf-5, Pf0-1, SBW25, and WH6**.

Table S2 **Links between Pseudomonas protein (“NCBI Protein Accession”), best hit to KEGG annotated transporter protein (“Best Hit,” “***e***-Value,” and “% Coverage”), with KEGG Orthology annotation (“KO Transporter”), and transported ligands (columns of ligand ID's with “1” indicating transport)**.

Table S3 **Table of aspen seedling phenotypic data**.

Table S4 **Loading table data for PCA analysis**.

Table S5 **Table of PRTT scores for Pseudomonads Pf-5, Pf0-1, SBW25, and WH6**.

Table S6 **Table of complete phenotype-transportome correlation network**.

## Abbreviations

CBD: Cumulative Binomial Distribution
Chl: Chlorophyll
DW: Dry Weight
EC: Enzyme Commission
GO: Gene Ontology
HCL: Hierarchical Clustering
KEGG: Kyoto Encyclopedia of Genes and Genomes
KO: KEGG Ontology
N: Nitrogen
OUT: Operational Taxonomic Unit
P: Phosphorus
PCA: Principal Component Analysis
PGP: Plant Growth Promotion
PRTT: Predicted Relative Transmembrane Transport.
