# Supplementary figures and images for: Pseudomonas fluorescens Transportome Is Linked to Strain-Specific Plant Growth Promotion in Aspen Seedlings under Nutrient Stress
Source: Front Plant Sci. 2017 Mar 21;8:348. doi: 10.3389/fpls.2017.00348 (PMC5359307; doi:10.3389/fpls.2017.00348)

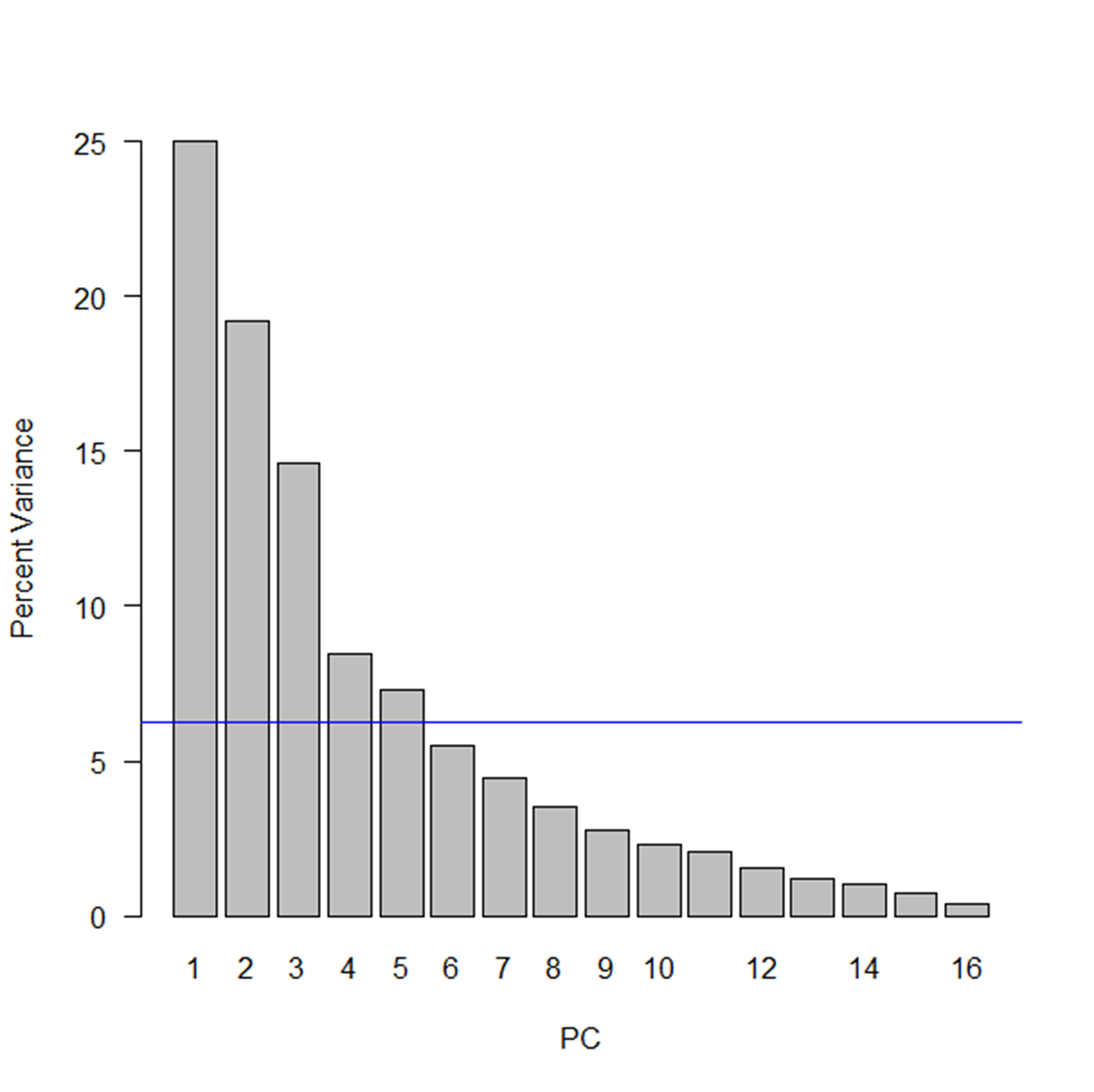

Supplement: Figure S1 — Bar graphs of amount of variance each PC contributes to PCA. [file Image1.TIFF]
